# Breast Cancer: Habitat imaging based on intravoxel incoherent motion for predicting pathologic complete response to neoadjuvant chemotherapy

**DOI:** 10.1002/mp.17813

**Published:** 2025-04-11

**Authors:** Hui Zhang, Yunyan Zheng, Mingzhe Zhang, Ailing Wang, Yang Song, Chenglong Wang, Guang Yang, Mingping Ma, Muzhen He

**Affiliations:** ^1^ Shengli Clinical College of Fujian Medical University & Department of Surgical Oncology, Fujian Provincial Hospital Fuzhou University Affiliated Provincial Hospital Fuzhou China; ^2^ Shengli Clinical College of Fujian Medical University & Department of Radiology, Fujian Provincial Hospital Fuzhou University Affiliated Provincial Hospital Fuzhou China; ^3^ Shanghai Key Laboratory of Magnetic Resonance East China Normal University Shanghai China; ^4^ MR Scientific Marketing Siemens Healthineers Ltd. Shanghai China

**Keywords:** breast cancer, habitat, intravoxel incoherent motion (IVIM), neoadjuvant chemotherapy (NAC), radiomics

## Abstract

**Background:**

Radiomics research based on whole tumors is limited by the unclear biological significance of radiomics features, which therefore lack clinical interpretability.

**Purpose:**

We aimed to determine whether features extracted from subregions defined by habitat imaging, reflecting tumor heterogeneity, could identify breast cancer patients who will benefit from neoadjuvant chemotherapy (NAC), to optimize treatment.

**Methods:**

143 women with stage II–III breast cancer were divided into a training set (100 patients, 36 with pathologic complete response [pCR]) and a test set (43 patients, 16 with pCR). Patients underwent 3‐T magnetic resonance imaging (MRI) before NAC. With the pathological results as the gold standard, we used the training set to build models for predicting pCR based on whole‐tumor radiomics (Model_WH_), intravoxel incoherent motion (IVIM)‐based habitat imaging (Model_Habitats_), conventional MRI features (Model_CF_), and immunohistochemical findings (Model_IHC_). We also built the combined models Model_Habitats+CF_ and Model_Habitats+CF+IHC_. In the test set, we compared the performance of the combined models with that of the invasive Model_IHC_ by using the area under the receiver operating characteristic curve (AUC) and decision curve analysis (DCA). Receiver operating characteristic (ROC) curve analysis was performed to evaluate the predictive value of the model. The DeLong test was used to compare diagnostic efficiency across different parameters.

**Results:**

In the prediction of pCR, Model_WH_, Model_Habitats_, Model_CF_, Model_IHC_, Model_Habitats+CF_, Model_CF+IHC_ and Model_Habitats+CF+IHC_ achieved AUCs of 0.895, 0.757, 0.705, 0.807, 0.800, 0.856, and 0.891 respectively, in the training set and 0.549, 0.708, 0.700, 0.788, 0.745, 0.909, and 0.891 respectively, in the test set. The DeLong test revealed no significant difference between Model_IHC_ versus Model_Habitats+CF_ (*p* = 0.695) and Model_Habitats+CF+IHC_ versus Model_CF+IHC_ (*p* = 0.382) but showed a significant difference between Model_IHC_ and Model_Habitats+CF+IHC_ (*p* = 0.043).

**Conclusion:**

The habitat model we established from first‐order features combined with conventional MRI features and IHC findings accurately predicted pCR before NAC. This model can facilitate decision‐making during individualized treatment for breast cancer.

**Abbreviations**
AUC: the area under the receiver operating characteristic curve
CF: conventional MRI‐features
D*: incoherent perfusion‐related microcirculation
D: pure diffusion coefficient
f: microvascular perfusion fraction
IHC: immunohistochemical
IVIM: Intravoxel incoherent motion
NAC: neoadjuvant chemotherapy
pCR: pathologic complete response
WH: whole‐tumor

## 1 BACKGROUND

According to the 2020 GLOBOCAN data, breast cancer has surpassed lung cancer to become the most commonly diagnosed cancer worldwide.^1^ Currently, the primary treatment for locally advanced breast cancer is neoadjuvant chemotherapy (NAC), which aims to shrink the primary tumor and downstage the disease, thereby facilitating breast conservation.^2^, ^3^ Breast cancer patients who attain a pathologic complete response (pCR) following NAC exhibit reduced rates of distant recurrence and experience extended periods of disease‐free survival.^4^, ^5^, ^6^ However, the reported rates of pCR after NAC in breast cancer patients vary widely from 6% to 45%.^7^, ^8^, ^9^, ^10^ Therefore, the timely identification of patients who will not respond to NAC can help them to avoid ineffective therapies and allow their treatment plans to be personalized. The response of breast cancer to NAC is recommended to be evaluated using magnetic resonance imaging (MRI), according to the National Comprehensive Cancer Network (NCCN) Clinical Practice Guidelines in Oncology‐Breast Cancer (v2.2023).

Radiomics has been widely applied for the analysis of malignant tumors, including analyses of gene mutations, protein expression, tissue typing and staging, treatment efficacy, prognosis, and survival, as well as for the formulation of individualized treatments.^11^, ^12^, ^13^ However, typical radiomics studies often suffer from lacking a clear biological meaning of radiomics features, and thence lack of clinical interpretability.^14^ Texture analysis is used to quantify spatial heterogeneity. However, it is often applied to the entire tumor, assuming that although the tumor is heterogeneous, it is “well mixed.” Thus, texture analysis neglects regional phenotypic variations within the tumor and fails to completely quantify intra‐tumor heterogeneity.^15^ For better analysis of intra‐tumor heterogeneity, a new approach, named habitat imaging or habitat analysis, was proposed, which uses multiparametric imaging data to divide tumors into subregions (habitats). The subregions or habitats correspond to microscopic clusters of cells with similar genotypes and phenotypes.^16^, ^17^

Intravoxel incoherent motion (IVIM) imaging has been proposed as a way of acquiring both diffusion and perfusion information in a completely noninvasive manner with a single imaging sequence.^18^ In recent years, some studies have used IVIM imaging to analyze areas of heterogeneity and hypoxia within the tumor.^19^, ^20^ IVIM can be analyzed using a biexponential model to simultaneously obtain perfusion parameters, including microvascular perfusion fraction (f), incoherent perfusion‐related microcirculation (D*), and pure diffusion coefficient (D).^18^ Since the biexponential IVIM model can yield parameters describing both the perfusion and diffusion within each voxel, these parameters can be used to cluster tumor voxels to yield habitats with different perfusion and diffusion status. This study aims to explore the value of IVIM‐habitat imaging in predicting pCR before NAC for breast cancer.

## 2 MATERIALS AND METHODS

### 2.1 Study setting and timeframe

**FIGURE 1:**
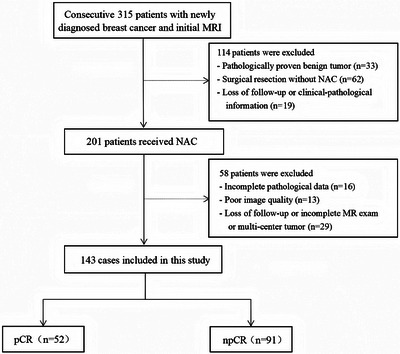
Flowchart depicting patient selection and grouping.

This prospective study consecutively enrolled patients with suspected breast cancer who underwent treatment at Fujian Provincial Hospital, between July 2019 and August 2023. The ethics committee of Fujian Provincial Hospital approved the study protocol (approval code: K2021‐05‐007, May 2019), and all methods in this study were carried out in accordance with relevant guidelines and regulations.^21^ Written informed consent to participate was obtained from all the patients. The inclusion criteria were: (I) no needle biopsy, radiotherapy, or chemotherapy before MRI examination; (II) availability of complete MRI review data before NAC with good image quality; (III) availability of complete pathological data; and (IV) without multicentric tumor. The process of patient selection and grouping has been illustrated in Figure 1.

### 2.2 Interventions

All included patients were treated with an NAC regimen formulated according to our institution's standards.^21^ Each enrolled patient underwent 6–8 cycles of intravenous NAC, over a period of 4–6 months, with each cycle lasting for 3 weeks. Following NAC, the patients underwent surgical removal of the tumor and regional lymph nodes. Total mastectomy or breast‐conserving surgery and axillary lymph node dissection were selected, according to the condition of the patient.

### 2.3 Immunohistochemical analysis

The expression of estrogen receptor (ER), progesterone receptor (PR), and human epidermal growth factor receptor 2 (HER2) expression, and the Ki67 index in the tumor tissue were determined using immunohistochemical (IHC) examination (Supplementary Material 1). The tissue samples for the IHC examination were collected using core needle biopsy, which was performed before NAC within 1 week after the MRI examination.

### 2.4 NAC

The distribution of NAC regimens in the included patients is shown in Supplementary Material 2. The NAC regimens were formulated according to the guidelines of the Chinese Society of Clinical Oncology (CSCO), from 2019 to 2023. After the completion of 6–8 cycles of NAC, the patients underwent surgical resection of the breast tumor tissue and regional lymph nodes. The surgically resected tissue samples were fixed, paraffin‐embedded, sliced into thin sections, and stained with hematoxylin and eosin. A pathologist (Y.H.) with 20 years of experience in diagnosing breast tumors was responsible for examining the tissues. The condition of the residual tumor in the resected surgical specimens was observed. Based on the guidelines of the CSCO, pCR was characterized as the lack of invasive carcinoma at the primary tumor site (with the possibility of ductal carcinoma in situ) and negative regional lymph nodes.

### 2.5 Imaging studies

MRI was conducted using a 3T scanner (MAGNETOM Prisma, Siemens Healthcare, Erlangen, Germany) equipped with an 18‐channel dual‐breast‐dedicated phase array surface coil. Patients were scanned in a prone position, allowing the breasts to be naturally positioned within the coil. The sequences encompassed in the imaging protocol are presented in Table 1. For the contrast‐enhanced scans, patients were administered gadopentetate meglumine (Magnevist, 0.2 mmol/kg; GE Healthcare). The contrast agent was injected into a dorsal vein of the hand via a high‐pressure syringe at a rate of 1.5–2.0 mL/s; this was followed by flushing with 15–20 mL of normal saline to clear any residual contrast agent. T1WI, fat saturation‐T2WI, and DCE‐MRI were used to comprehensively evaluate the conventional MRI features.

**TABLE 1:** Imaging protocol of MR sequences.

	TR	TE	Thickness	Slices	Bandwidth	Matrix	Averages	Concatenations	FOV	Scan time	Others
Fat saturation T2WI	3739 ms	69 ms	4 mm	35	246 Hz/Px	384 × 384	2	2	340 × 340 mm	157 s	/
T1WI	6.03 ms	2.82 ms	0.9 mm	160	300 Hz/Px	403 × 448	1	1	340 × 340 mm	117 s	/
DCE‐MRI	4.03 ms	1.33 ms	1.5 mm	112	1120 Hz/Px	259 × 320	1	1	350 × 350 mm	343 s	Measurements 36
DWI	5700 ms	62 ms	4 mm	35	2024 Hz/Px	114 × 190	1, 2, 2, 2, 2, 2, 2, 2, and 3	1	340 × 340 mm	318 s	*B*‐values 0, 30, 50, 80, 120, 160, 200, 500, and 1000 s/mm^2^

### 2.6 Tumor segmentation

To obtain IVIM maps, we carried out a pixel‐by‐pixel fitting of the diffusion‐weighted imaging data by using the prototype research software Body Diffusion Toolbox (Siemens Healthcare, Erlangen, Germany). This software generated maps of IVIM parameters, including D, f, and D*. The IVIM images were visualized using 3D Slicer (*v*4.10.2, www.slicer.org) for image segmentation. A radiologist (M.H.) with 14 years of experience in breast imaging diagnosis performed the segmentations. A three‐dimensional region of interest (ROI) that included the solid tumor component was drawn on IVIM‐D maps, and for lesion edges with suboptimal contrast, we referred to the DCE map to determine the edges. The MRI scans of the patients were randomly divided into the training (*n* = 100 patients) and test sets (*n* = 43 patients). Another radiologist (Y.Z.) with 5 years of experience in breast imaging independently segmented 30 randomly selected tumors from the training set. The interobserver reproducibility was assessed using the two‐way random absolute agreement intraclass correlation coefficient (ICC). The ICC values ranged from 0 to 1 and were interpreted as follows: ICC ≥ 0.75, good consistency; 0.50 ≤ ICC < 0.75, general consistency; and ICC < 0.50, poor consistency. Additionally, we combined the different quantitative maps (D, f, and D*) and applied K‐means, an unsupervised clustering method, to split voxels in the whole‐tumor ROI into three parts. The details are presented in Supplementary Material 3.

### 2.7 Habitat analysis and feature extraction

We used PyRadiomics (*v*3.0)^22^ to extract quantitative features from an ROI that encompassed the whole tumor. We tried to determine the number of habitats using a bootstrap approach (the details are presented in Supplementary Material 4). In our study, we employed a two‐stage feature selection approach. For each part, we extracted the volume, the volume ratio, and the first‐order features from different quantitative maps. These features were used to describe tumor heterogeneity.

### 2.8 Model development

We excluded features with ICCs < 0.75; the remaining features were considered to be stable features. In both the training and the test set, we normalized the selected features by *z*‐score, which is calcualted using the following formula:

$$y=\frac{x-\mathrm{mean}\left(x\right)}{\mathrm{std}\left(x\right)}$$

where both mean values and standard deviations were calculated using only the training set. We applied the Pearson correlation coefficient (cutoff = 0.9) between each pair of features to reduce the dimensionality of the feature space. Next, we applied the least absolute shrinkage and selection operator (LASSO) regression to remove redundant features through regularization. Subsequently, we utilized recursive feature elimination (RFE) to iteratively select the most relevant features. The rationale for this approach is that starting with RFE alone can be computationally expensive and prone to the influence of redundant features, potentially leading to suboptimal feature selection. By first applying LASSO, we mitigated these issues by reducing the feature space, thereby improving the efficiency and effectiveness of the RFE process.^23^, ^24^ The LASSO regularization parameter (alpha) was optimized within an exponentially spaced range (0.005–0.05) using 5‐fold cross‐validation (LassoCV) with extended iterations and relaxed tolerance to balance feature selection and numerical stability. For RFE, we iteratively removed the features until the determined number of features remained. The classifier, which includes support vector machine and logistic regression (the details are presented in Supplementary Material 5), was utilized to effectively predict outcomes based on the features extracted from the datasets. The performance of the selected features was assessed using 5‐fold cross‐validation in the training set.

**FIGURE 2:**
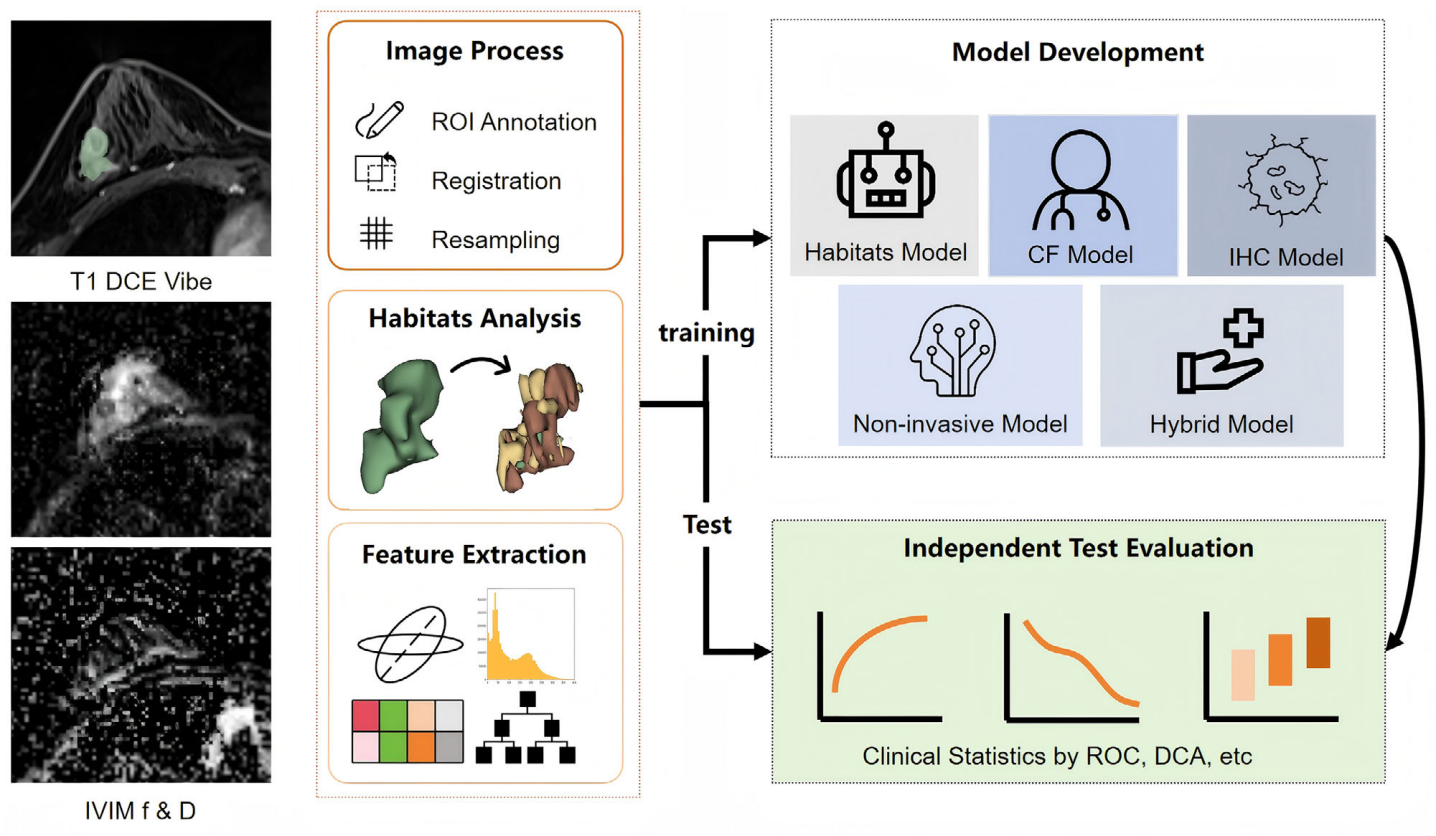
Workflow for the establishment of the models.

Then, we built two IVIM‐based prediction models: a model based on features extracted from whole‐tumor ROIs (Model_WH_) and a model based on features extracted from tumor habitats (Model_Habitats_). Both IVIM‐based models were constructed using the above process. We selected different parameter‐map combinations to build Model_Habitats_ based on the best performance of the model; the details are presented in Supplementary Material 3. Breast lesions were evaluated for conventional MRI signs based on the 2013 Breast Imaging—Reporting and Data System (BI‐RADS) for MRI. We analyzed 11 conventional MRI features for each patient: tumor size, amount of fibroglandular tissue, background parenchymal enhancement level, T2 signal intensity, masses or non‐mass enhancement, tumor shape, tumor margin, internal enhancement characteristics, non‐mass internal enhancement pattern, architectural distortion, and time‐intensity curve. We also built a prediction model based on conventional MRI features (Model_CF_) and another model based on IHC features (Model_IHC_), and trained them using logistic regression. Next, we combined the selected features to build the non‐invasive Model_Habitats+CF_ and the invasive combined Model_CF+IHC_, Model_Habitats+CF+IHC_. The performance of all these models in predicting pCR was evaluated in the independent test set (Figure 2).

### 2.9 Statistical analysis

Statistical analysis was performed using SPSS *v*28.0.1.1. Continuous variables were evaluated for normal distribution and homogeneity of variance by using the Shapiro–Wilk test and the Levene variance test, respectively. Continuous variables that showed a normal distribution were presented as mean and standard deviation, and compared between groups by using the two independent samples *t*‐test. Continuous variables with a skewed distribution were expressed as median (interquartile range) and analyzed using the Mann–Whitney *U* test. Categorical variables were expressed as frequency (percentage) and compared between groups by using the Pearson Chi‐square test and the continuous‐corrected Chi‐square test. With the pathological results as the gold standard, we performed receiver operating characteristic (ROC) curve analysis to evaluate the predictive value of the model and calculated the area under the ROC curve (AUC). The cutoff AUC value was chosen according to the maximum Youden index. The predictive ability of the models was evaluated using ROC curves and various classification measures, including AUC, sensitivity, specificity, positive predictive value (PPV), negative predictive value (NPV), accuracy, and Matthews correlation coefficient. The DeLong test was used to compare diagnostic efficiency across different parameters. Decision curve analysis (DCA) was employed to plot the net benefit rate against the threshold of PCR prediction. *p* (two‐tailed) < 0.05 was considered statistically significant.

## 3 RESULTS

### 3.1 Dataset segmentation

After the application of the selection criteria, the present prospective study included a total of 143 women diagnosed with stage II–III invasive ductal carcinoma of the breast, and pCR was attained in 52 of these women. The patients were randomly split into a training set with 100 patients (36 with pCR) and a test set with 43 patients (16 with pCR). No significant differences (*p* > 0.05) in IHC expression were found between the training and test sets (Table 2).

**TABLE 2:** Characteristics of patients in the training and test sets (*n* = 143).

Characteristic	Training set (*n* = 100)	Test set (*n* = 43)	*p*‐value
Age (years), mean ± SD	48.5 ± 9.6	50.0 ± 10.7	0.675
Histological type, *n* (%)			0.760
Ductal	91 (91)	38 (88)	
Others	9 (9)	5 (12)	
HR status, *n* (%)			0.345
Positive	62 (62)	22 (51)	
Negative	38 (38)	21 (49)	
HER2 status, *n* (%)			0.716
Positive	49 (49)	23 (53)	
Negative	51 (51)	20 (47)	
Ki67 index, *n* (%)			0.823
High	78 (78)	35 (81)	
Low	22 (22)	8 (19)	
Lymph nodes, *n* (%)			0.060
Positive	71 (71)	37 (86)	
Negative	29 (29)	6 (14)	
pCR			1.000
Yes	36 (36)	16 (37)	
No	64 (64)	27 (63)	

*Note*: Only variables with *p* < 0.05 were identified as significant.

Abbreviations: HER2, human epidermal growth factor receptor 2; HR, hormone receptor; pCR, pathologic complete response.

### 3.2 Performance of the single‐modality models

**FIGURE 3:**
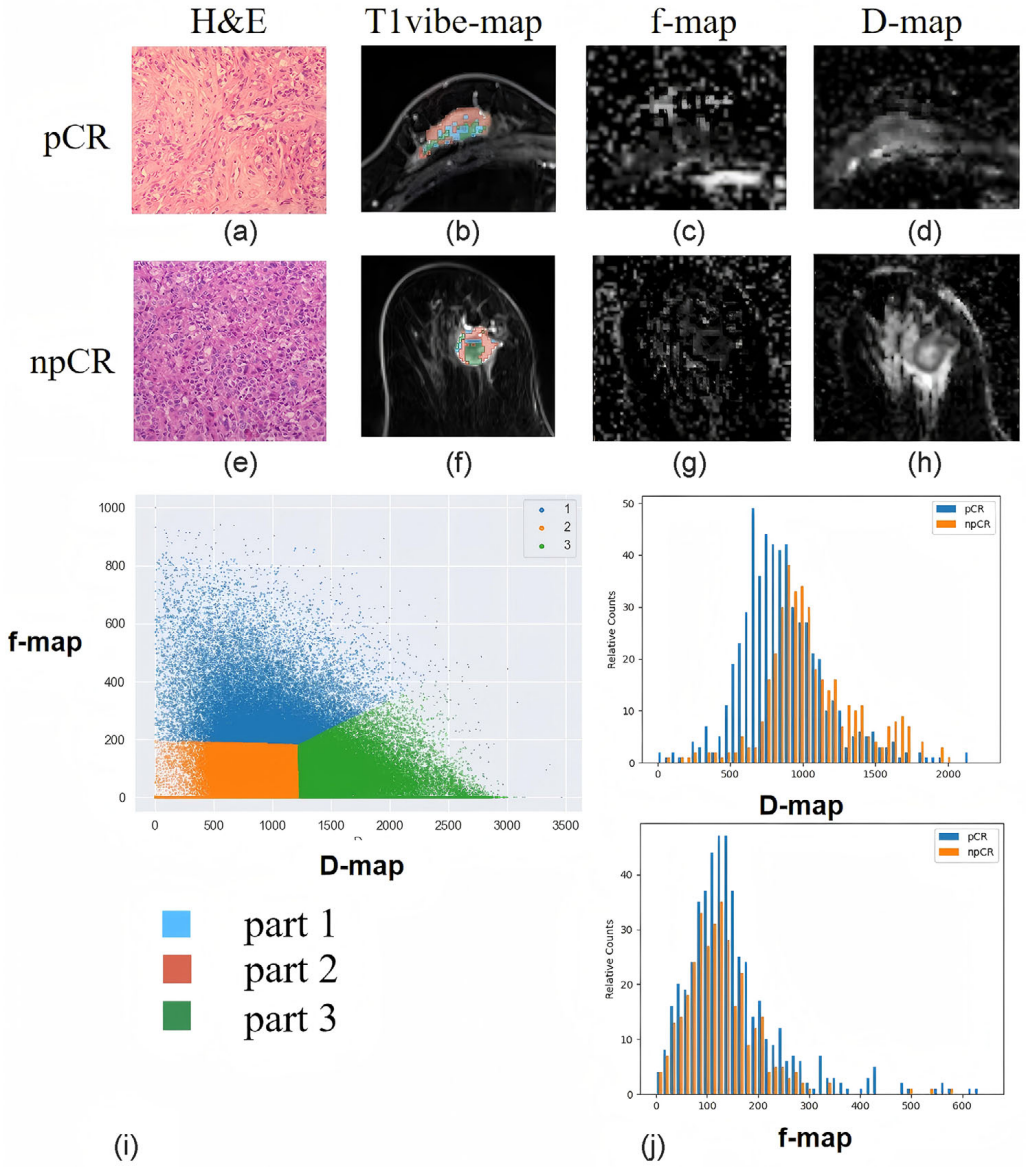
Habitat imaging of patients with and without pCR following neoadjuvant chemotherapy. (a,e) Hematoxylin‐eosin staining (×100). (b,f) Habitat imaging. (c,g) f map. (d,h) D map. (i) Voxel distribution in regions of interest in the D and f maps (*K* = 3). Part 1 stands for the region of high *f*‐value, representing high perfusion; part 2 stands for region of low *D*‐value and low *f*‐value, representing high cell density; and part 3 stands for region of high *D*‐value and low *f*‐value, representing low cell density and low perfusion. The dominant regions within tumors with pCR were areas of high perfusion and high cell density, while areas with low perfusion and low cell density dominated within tumors without pCR. (j) Histograms of D maps and f maps obtained from the pCR and non‐pCR groups. pCR, pathologic complete response.

In total, 1235 features were extracted from each sequence. The AUCs of Model_WH_ in the training and testing sets were 0.895 and 0.549, respectively (Table 3). For Model_Habitats_, the best model that using f and D maps with clustering *K* = 3 achieved the highest AUC on the validation. The AUC of this model was 0.757 in the training set and 0.708 in the test set. The whole‐tumor ROI was split into a high‐f region, a low‐f low‐D region, and a low‐f high‐D region (Figure 3e). The conventional MRI features of each patient were analyzed (Table 4). Model_CF_ was built using the features rim enhancement and high T2 signal, and showed AUCs of 0.705 in the training set and 0.700 in the test set (Table 3).

**TABLE 3:** Results of ROC curve analysis for predicting pCR using the whole‐tumor radiomic model, the habitat model, the conventional MRI features model, the immunohistochemical model, and the combined models before NAC.

Model	AUC (95% CI)	Accuracy	Balanced accuracy	Sensitivity	Specificity	PPV	NPV	Matthews correlation coefficient
Whole‐tumor radiomics								
Training set	0.895 (0.834, 0.950)	0.780	0.816	0.944	0.688	0.630	0.957	0.609
Test set	0.549 (0.356, 0.748)	0.488	0.504	0.563	0.444	0.375	0.632	0.007
Habitat analysis								
Training set	0.757 (0.653, 0.848)	0.720	0.714	0.694	0.734	0.595	0.810	0.417
Test set	0.708 (0.543, 0.859)	0.674	0.639	0.500	0.778	0.571	0.724	0.287
Conventional MRI features								
Training set	0.705 (0.549, 0.860)	0.651	0.685	0.813	0.556	0.520	0.833	0.361
Test set	0.700 (0.581, 0.810)	0.674	0.619	0.353	0.885	0.667	0.676	0.286
Immunohistochemistry								
Training set	0.807 (0.722, 0.892)	0.790	0.788	0.778	0.797	0.684	0.864	0.561
Test set	0.788 (0.657, 0.922)	0.733	0.679	0.469	0.889	0.714	0.738	0.402
Habitat + Conventional MRI features								
Training set	0.800 (0.705, 0.884)	0.760	0.746	0.694	0.797	0.658	0.823	0.486
Test set	0.745 (0.590, 0.901)	0.720	0.664	0.438	0.889	0.700	0.727	0.373
Whole‐tumor radiomics + Conventional MRI features								
Training set	0.843 (0.768, 0.919)	0.760	0.825	0.944	0.656	0.607	0.955	0.581
Test set	0.634 (0.465, 0.804)	0.581	0.578	0.563	0.593	0.450	0.696	0.150
Immunohistochemistry + Conventional MRI features								
Training set	0.856 (0.773, 0.939)	0.790	0.812	0.889	0.734	0.653	0.922	0.599
Test set	0.909 (0.826, 0.991)	0.791	0.796	0.813	0.778	0.684	0.875	0.575
Whole‐tumor radiomics + Conventional MRI features + Immunohistochemistry								
Training set	0.888 (0.825, 0.951)	0.820	0.817	0.806	0.828	0.725	0.883	0.621
Test set	0.803 (0.667, 0.939)	0.767	0.752	0.688	0.815	0.688	0.815	0.502
Habitat + Conventional MRI features + Immunohistochemistry								
Training set	0.891 (0.821, 0.963)	0.820	0.842	0.917	0.766	0.688	0.942	0.656
Test set	0.891 (0.799, 0.984)	0.744	0.759	0.813	0.704	0.619	0.864	0.499

Abbreviations: AUC, area under the curve; CI, confidence interval; MRI, magnetic resonance imaging; NAC, neoadjuvant chemotherapy; NPV, negative predictive value; pCR, pathologic complete response; PPV, positive predictive value; ROC, receiver operating characteristic.

**TABLE 4:** Conventional MRI signs before NAC in 143 breast cancer patients.

Parameter	pCR (*n* = 52)	No pCR (*n* = 91)	*t*/*χ* ^2^	*p*‐value
Size (mm)	36.8 ± 8.7	41.8 ± 10.7	0.765	0.145
FGT			0.100	1.000
a/b	6 (12)	11 (12)		
c/d	46 (88)	80 (88)		
BPE			0.591	0.484
a/b	28 (54)	55 (60)		
c/d	24 (46)	36 (40)		
High T2 signal			11.204	0.001
Positive	9 (17)	41 (45)		
Negative	43 (83)	50 (55)		
Masses	32 (62)	49 (54)	0.797	0.387
Mass shape (%)			0.080	0.773
Round/Oval	6 (19)	8 (16)		
Irregular	26 (81)	41 (84)		
Mass margin (%)			0.583	0.493
Spiculated	11 (34)	21 (43)		
Non‐spiculated	21 (66)	28 (57)		
Internal enhancement pattern (%)			1.106	0.325
Rim enhancement	25 (78)	33 (67)		
No rim enhancement	7 (22)	16 (33)		
Non‐mass enhancement	20 (38)	42 (46)		
Non‐mass internal enhancement pattern (%)			0.004	1.000
Clustered ring	18 (90)	38 (90)		
Homogeneous/Heterogeneous	2 (10)	4 (10)		
Architectural distortion			0.002	1.000
Positive	11 (21)	19 (21)		
Negative	41 (79)	72 (79)		
Time‐intensity curve			2.085	0.258
I	1 (2)	7 (8)		
II/III	51 (98)	84 (92)		

*Note*: Only variables with *p* < 0.05 were identified as significant. FGT: a, Almost entirely fat;b, Scattered fibroglandular tissue; c, Heterogeneous fibroglandular tissue; d, Extreme fibroglandular tissue; BPE: a, Minimal; b, Mild; c, Moderate; d, Marked; Time‐intensity curve: I, Persistent; II, Plateau; III, Washout.

Abbreviations: BPE, background parenchymal enhancement; FGT, fibroglandular tissue; MRI, magnetic resonance imaging; NAC, neoadjuvant chemotherapy; pCR, pathologic complete response.

Pre‐NAC biopsy samples were used to determine the expression of HRs, HER2, and Ki67 on IHC for each patient. Model_IHC_ was built using the features HR negativity and HER2 positivity, and exhibited AUCs of 0.807 and 0.788 in the training and test set, respectively (Table 3).

### 3.3 Performance of the combined models

Model_Habitats+CF_ combined the results of habitat analysis based on the D and f maps with conventional MRI features. The AUCs of Model_Habitats+CF_, Model_CF+IHC_ were 0.800, 0.856 in the training sets and 0.745, 0.909 in the test sets, respectively (Table 3). Model_Habitats+CF+IHC_ combined habitat analysis, conventional MRI features, and IHC findings. The AUCs of this model in the training and test sets were 0.891 and 0.891, respectively (Table 3). The details of the final features and the importance of each of these variables in the combined models are presented in Supplementary Material 6.

### 3.4 Model comparison

#### 3.4.1 Model_WH_ versus Model_Habitats_

The DeLong test indicated significant differences between the model combining the results of habitat analysis based on D and *f*‐values, and the model based on whole‐tumor radiomics analysis (*p*‐values: 0.00908).

#### 3.4.2 Model_IHC_ versus Model_Habitats+CF_ versus Model_Habitats+CF+IHC_ and Model_Habitats+CF+IHC_ versus Model_CF+IHC_

**FIGURE 4:**
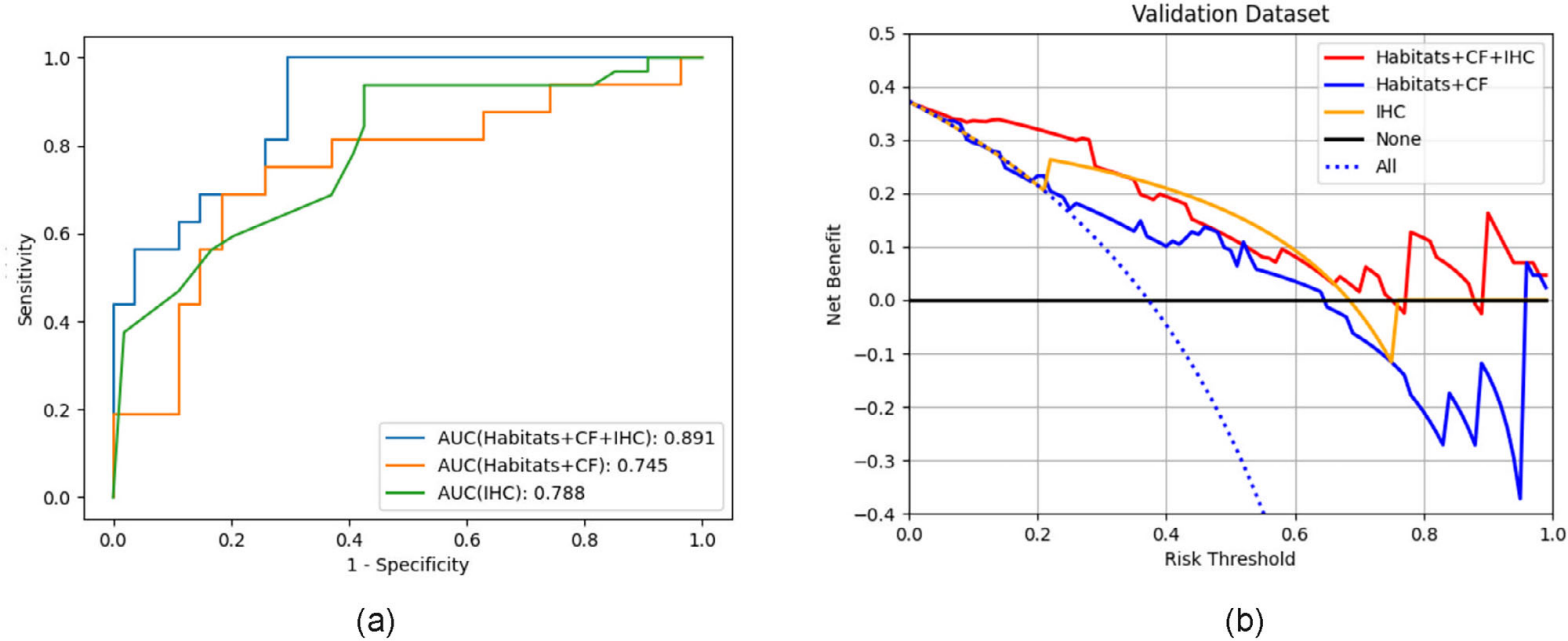
(a) ROC curves for pCR prediction models. (b) DCA of the performance of the models. ROC, receiver operating characteristic. DCA, decision curve analysis; pCR, pathologic complete response; ROC, receiver operating characteristic. AUC, area under the curve; CF, conventional features; IHC, immunohistochemistry

The DeLong test showed no significant difference between the performance of Model_IHC_ versus Model_Habitats+CF_ (*p* = 0.695) and Model_Habitats+CF+IHC_ versus Model_CF+IHC_ (*p* = 0.382). However, a significant difference was observed when Model_IHC_ was compared to Model_Habitats+CF+IHC_ (*p* = 0.043). Model_Habitats+CF+IHC_ provided a greater net benefit for clinical intervention than did all the other models. Additionally, DCA^25^ indicated that the different models had favorable clinical utility (Figure 4).

## 4 DISCUSSION

In this study, a model built using IVIM‐based habitat analysis and conventional MRI features demonstrated comparable efficacy to that of a model based on invasive biopsies (Model_IHC_) in predicting pCR after NAC for locally advanced breast cancer. Habitat analysis could quantitatively assess the spatiotemporal heterogeneity of the entire tumor, thereby achieving a “virtual biopsy”. All patients were pathologically diagnosed with invasive ductal carcinoma in our study; thus, the patient type was uniform and potentially not a confounding factor. CSCO guidelines recommend choosing different NAC regimens according to different breast cancer IHC findings. Our model jointly considers both IHC and intra‐tumor heterogeneity analysis using habitat imaging to predict the efficacy of NAC. The model combining pre‐NAC IVIM‐based habitat analysis, conventional MRI features, and molecular markers (Model_Habitats+CF+IHC_) achieved an AUC of 0.891 in predicting pCR. This suggests that the underlying biological features captured by the imaging may supplement the specificities of the treatment protocols. The model combining habitat imaging with IHC markers significantly outperformed the IHC model in terms of predictive efficacy. Additionally, when comparing the model based on habitat analysis with that based on whole‐tumor radiomics, we found that habitat analysis had superior predictive efficacy, and was more interpretable with histogram features. By constructing different habitats (subregions) within the tumor through habitat analysis, we discovered that tumor areas with high vascular perfusion and high cell density were more likely to achieve pCR after NAC. The IVIM‐based habitat model was not only an effective predictive model but also identified the biological significance of the radiomic features.

The precise prediction of post‐NAC pCR is vital for accurate clinical decision‐making and prognostication for breast cancer patients. In recent years, multiple studies have demonstrated that radiomics holds great potential in predicting the efficacy of NAC for breast cancer.^26^, ^27^ However, the biological interpretability of the extracted radiomic features requires further exploration. IVIM parameters can reflect tumor perfusion and water molecule diffusion, enabling the construction of clustered subregions, such as areas with high perfusion and high cell density, high perfusion and low cell density, low perfusion and low cell density, and low perfusion and high cell density. Our research found that within a tumor, areas with high *f*‐values and low *D*‐values, as constructed using IVIM, are more likely to achieve pCR, while areas with low *f*‐values and high *D*‐values showed a poorer response to NAC. Consistent with previous findings, lower pre‐NAC *D*‐values were observed in good responders.^28^ Feng et al.^29^ also found that the *f*‐value might predict the expression of HER2. HER2+ expression can induce the generation of VEGF, which promotes the proliferation of cancer cells, requiring targeting drugs for treatment. Patients with the HER2 subtype of breast cancer are more likely to achieve post‐treatment pCR.^30^ Areas with high *D*‐values represent necrotic tissue with low cellularity.^31^ The reason is that areas with high cell proliferation have increased cell density, which reduces the extracellular volume, thereby restricting water molecule diffusion and reducing the *D*‐value. In contrast, necrotic tumor tissue is likely in a hypoxic environment and has a slow metabolism, and therefore, is less sensitive to chemotherapeutic agents.^31^ Hence, tumor areas with high cell proliferation or density and high perfusion are likely beneficial in terms of drug delivery. The heterogeneity we identified using baseline MRI‐IVIM habitat imaging suggests that different areas of the tumor may respond variably to treatment, and regions with specific imaging markers are more likely to respond effectively to NAC. The habitat model was more explanatory than the whole‐tumor‐radiomics analysis. The habitat model's focus on localized regions enhances its ability to capture more specific patterns. By zooming in on particular tumor areas, the model improves its sensitivity to changes in local tissue properties, which might be diluted or averaged out in the whole‐tumor approach. Therefore, the technical advantages of the habitat model, such as localized feature extraction, better handling of tumor heterogeneity, multiscale analysis, and improved model robustness, make it superior to the conventional whole‐tumor analysis model, particularly when considering the complexity of tumor microenvironments and the need for personalized treatment strategies.^16^, ^17^, ^32^, ^33^

Among the CF of MRI, we selected rim enhancement and high T2 signal to build Model_CF_ using logistic regression. Studies have indicated that triple‐negative breast cancer is more likely to exhibit rim enhancement and achieve pCR following NAC.^34^, ^35^, ^36^ The presence of a high T2 signal within the lesion is associated with tumor necrosis. Consistent with previous research,^37^, ^38^ our study showed that HR‐negative and HER2‐positive tumors on pre‐NAC biopsy were more inclined towards achieving pCR. The DeLong test result (*p *= 0.382) indicates that the difference in AUC between Model_Habitats+CF+IHC_ and Model_CF+IHC_ is not statistically significant. This suggests that the observed AUC improvement is likely due to random variation rather than a true enhancement in model performance. Therefore, from a purely AUC‐based perspective, the inclusion of Model_Habitats_ does not lead to a statistically significant improvement, at least over the current dataset. However, Model_Habitats_ may still contribute additional biological insights. Compared to a model relying solely on CF and IHC features, Model_Habitats_ may using the multi‐parametric MR images and capture spatial information, tissue microenvironment characteristics, and local heterogeneity, where treatment resistance arises from spatially heterogeneous microenvironments. While these features may not translate into a substantial gain in AUC, they may enhance model interpretability, improve individualized risk assessment, and provide better predictions for specific patient subgroups. Thus, despite the limited overall performance gain, Model_Habitats_ still hold value in specific clinical applications or pathological analyses. In this study, Model_Habitat+CF+IHC_ generalized well. The features of Model_Habitat+CF+IHC_ were derived from both Model_IHC_ and Model_Habitat+CF_. The combination of features from Model_IHC_ and Model_Habitat+CF_ may create a more comprehensive representation of the data. This synergy can capture more relevant patterns and relationships, improving the model's ability to generalize to the test set. By integrating features from two different models, Model_Habitat+CF+IHC_ might benefit from a wider range of information. This diversity can enhance its robustness against variations in the test data, leading to better performance.

### 4.1 Limitations

We must acknowledge the limitations of our study. First, while we have explored the potential of a noninvasive habitat‐based approach in our study, we acknowledge the need for further validation based on larger cohorts. Secondly, patients treated with different treatment regimens were included to build a single model in our study. A more accurate stratified study can be performed when enough data can be collected. Our model was developed using a single‐center cohort without external validation. However, it is noteworthy that in our habitat model, we only extracted first‐order features, which can potentially increase the generalizability of the model, although this needs to be further validated in external datasets. The second‐order features in radiomics are generally more sensitive to variations in MRI machines and parameters, as they capture spatial relationships and texture patterns, which are affected by resolution, noise, and scanning protocols. The subjectiveness of manual segmentation may also negatively influence the stability of the model, which can be resolved by training an automatic segmentation model in a future study. Additionally, the habitat model took into consideration the spatial heterogeneity of the tumor. The quantitative sequence model (Model_Habitats_) is amenable to broader application and generalization across different MR machines. While we performed habitat imaging of the whole tumor, routine clinical biopsy examination cannot obtain full‐size tissue sections corresponding to the imaging data, due to spatial heterogeneity. Hence, the co‐registration of pathology slices with the imaging scans of the same layer to further interpret the biological information suggested by imaging merits further exploration.

## 5 CONCLUSION

In conclusion, the predictive efficacy of the habitat model surpassed that of the whole‐tumor radiomic model, with the habitat model incorporating an analysis of the spatial heterogeneity of the tumor. The non‐invasive habitat model was comparable in efficacy to the invasive IHC model, which requires pathology. The habitat model we established from first‐order features combined with conventional MRI features and IHC findings could accurately predict pCR before NAC. Hence, it can serve as a valuable tool for informing decision‐making in the individualized treatment of breast cancer patients.

## CONFLICT OF INTEREST STATEMENT

The authors declare no conflicts of interest.

## Supporting information



Supporting Information

Supporting Information

Supporting Information

Supporting Information

## Data Availability

The data that support the findings of this study are not publicly available due to limitations of ethical approval involving the patient data and anonymity but are available from the corresponding author on reasonable request.

## References

[1] Sung H , Ferlay J , Siegel RL , et al. Global cancer statistics 2020: GLOBOCAN estimates of incidence and mortality worldwide for 36 cancers in 185 countries. CA Cancer J Clin. 2021;71(3):209‐249. doi:10.3322/caac.21660 33538338

[2] Burstein HJ , Curigliano G , Thürlimann B , et al. Customizing local and systemic therapies for women with early breast cancer: the St. Gallen International Consensus Guidelines for treatment of early breast cancer 2021. Ann Oncol. 2021;32(10):1216‐1235. doi:10.1016/j.annonc.2021.06.023 34242744 PMC9906308

[3] Halberg AK , Gravesen CD , Cold S , Jensen JD . Neoadjuvant chemotherapy for primary operable breast cancer. Dan Med J. 2020;67(12):A01200010. 33269690

[4] Cortazar P , Zhang L , Untch M , et al. Pathological complete response and long‐term clinical benefit in breast cancer: the CTNeoBC pooled analysis. Lancet. 2014;384(9938):164‐172. doi:10.1016/s0140-6736(13)62422-8 24529560

[5] Early Breast Cancer Trialists' Collaborative Group (EBCTCG) . Long‐term outcomes for neoadjuvant versus adjuvant chemotherapy in early breast cancer: meta‐analysis of individual patient data from ten randomised trials. Lancet Oncol. 2018;19(1):27‐39. doi:10.1016/s1470-2045(17)30777-5 29242041 PMC5757427

[6] Eremin J , Cowley G , Walker LG , Murray E , Stovickova M , Eremin O . Women with large (≥3 cm) and locally advanced breast cancers (T3, 4, N1, 2, M0) receiving neoadjuvant chemotherapy (NAC: cyclophosphamide, doxorubicin, docetaxel): addition of capecitabine improves 4‐year disease‐free survival. Springerplus. 2015;4(1):9. doi:10.1186/2193-1801-4-9 25995984 PMC4429427

[7] Bonnefoi H , Litière S , Piccart M , et al. Pathological complete response after neoadjuvant chemotherapy is an independent predictive factor irrespective of simplified breast cancer intrinsic subtypes: a landmark and two‐step approach analyses from the EORTC 10994/BIG 1‐00 phase III trial. Ann Oncol. 2014;25(6):1128‐1136. doi:10.1093/annonc/mdu118 24618153 PMC4037859

[8] von Minckwitz G , Untch M , Blohmer JU , et al. Definition and impact of pathologic complete response on prognosis after neoadjuvant chemotherapy in various intrinsic breast cancer subtypes. J Clin Oncol. 2012;30(15):1796‐1804. doi:10.1200/jco.2011.38.8595 22508812

[9] Gonzalez‐Angulo AM , Morales‐Vasquez F , Hortobagyi GN . Overview of resistance to systemic therapy in patients with breast cancer. Adv Exp Med Biol. 2007;608:1‐22. doi:10.1007/978-0-387-74039-3_1 17993229

[10] Zambetti M , Mansutti M , Gomez P , et al. Pathological complete response rates following different neoadjuvant chemotherapy regimens for operable breast cancer according to ER status, in two parallel, randomized phase II trials with an adaptive study design (ECTO II). Breast Cancer Res Treat. 2012;132(3):843‐851. doi:10.1007/s10549-011-1660-6 21750964

[11] Aerts HJ . The potential of radiomic‐based phenotyping in precision medicine: a review. JAMA Oncol. 2016;2(12):1636‐1642. doi:10.1001/jamaoncol.2016.2631 27541161

[12] Lambin P , Leijenaar RTH , Deist TM , et al. Radiomics: the bridge between medical imaging and personalized medicine. Nat Rev Clin Oncol. 2017;14(12):749‐762. doi:10.1038/nrclinonc.2017.141 28975929

[13] Tagliafico AS , Piana M , Schenone D , Lai R , Massone AM , Houssami N . Overview of radiomics in breast cancer diagnosis and prognostication. Breast. 2020;49:74‐80. doi:10.1016/j.breast.2019.10.018 31739125 PMC7375670

[14] Yip SS , Aerts HJ . Applications and limitations of radiomics. Phys Med Biol. 2016;61(13):R150‐166. doi:10.1088/0031-9155/61/13/r150 27269645 PMC4927328

[15] Gatenby RA , Grove O , Gillies RJ . Quantitative imaging in cancer evolution and ecology. Radiology. 2013;269(1):8‐15. doi:10.1148/radiol.13122697 24062559 PMC3781355

[16] Chang YC , Ackerstaff E , Tschudi Y , et al. Delineation of tumor habitats based on dynamic contrast enhanced MRI. Sci Rep. 2017;7(1):9746. doi:10.1038/s41598-017-09932-5 28851989 PMC5575347

[17] Wu J , Cao G , Sun X , et al. Intratumoral spatial heterogeneity at perfusion MR imaging predicts recurrence‐free survival in locally advanced breast cancer treated with neoadjuvant chemotherapy. Radiology. 2018;288(1):26‐35. doi:10.1148/radiol.2018172462 29714680 PMC6029132

[18] Koh DM , Collins DJ , Orton MR . Intravoxel incoherent motion in body diffusion‐weighted MRI: reality and challenges. AJR Am J Roentgenol. 2011;196(6):1351‐1361. doi:10.2214/ajr.10.5515 21606299

[19] Jalnefjord O , Montelius M , Arvidsson J , Forssell‐Aronsson E , Starck G , Ljungberg M . Data‐driven identification of tumor subregions based on intravoxel incoherent motion reveals association with proliferative activity. Magn Reson Med. 2019;82(4):1480‐1490. doi:10.1002/mrm.27820 31081969 PMC6767386

[20] Hompland T , Hole KH , Ragnum HB , et al. Combined MR imaging of oxygen consumption and supply reveals tumor hypoxia and aggressiveness in prostate cancer patients. Cancer Res. 2018;78(16):4774‐4785. doi:10.1158/0008-5472.can-17-3806 29945958

[21] Li J , Jiang Z . Chinese Society of Clinical Oncology Breast Cancer (CSCO BC) guidelines in 2022: stratification and classification. Cancer Biol Med. 2022;19(6):769‐773. doi:10.20892/j.issn.2095-3941.2022.0277 35765123 PMC9257320

[22] van Griethuysen JJM , Fedorov A , Parmar C , et al. Computational radiomics system to decode the radiographic phenotype. Cancer Res. 2017;77(21):e104‐e107. doi:10.1158/0008-5472.can-17-0339 29092951 PMC5672828

[23] Huang YQ , Liang CH , He L , et al. Development and validation of a radiomics nomogram for preoperative prediction of lymph node metastasis in colorectal cancer. J Clin Oncol. 2016;34(18):2157‐2164. doi:10.1200/jco.2015.65.9128 27138577

[24] Zhang X , Xu X , Tian Q , et al. Radiomics assessment of bladder cancer grade using texture features from diffusion‐weighted imaging. J Magn Reson Imaging. 2017;46(5):1281‐1288. doi:10.1002/jmri.25669 28199039 PMC5557707

[25] Vickers AJ , Holland F . Decision curve analysis to evaluate the clinical benefit of prediction models. Spine J. 2021;21(10):1643‐1648. doi:10.1016/j.spinee.2021.02.024 33676020 PMC8413398

[26] Liu Z , Li Z , Qu J , et al. Radiomics of multiparametric MRI for pretreatment prediction of pathologic complete response to neoadjuvant chemotherapy in breast cancer: a multicenter study. Clin Cancer Res. 2019;25(12):3538‐3547. doi:10.1158/1078-0432.ccr-18-3190 30842125

[27] Guo L , Du S , Gao S , et al. Delta‐radiomics based on dynamic contrast‐enhanced MRI predicts pathologic complete response in breast cancer patients treated with neoadjuvant chemotherapy. Cancers. 2022;14(14):3515. doi:10.3390/cancers14143515 35884576 PMC9316501

[28] Bedair R , Priest AN , Patterson AJ , et al. Assessment of early treatment response to neoadjuvant chemotherapy in breast cancer using non‐mono‐exponential diffusion models: a feasibility study comparing the baseline and mid‐treatment MRI examinations. Eur Radiol. 2017;27(7):2726‐2736. doi:10.1007/s00330-016-4630-x 27798751 PMC5486805

[29] Feng W , Gao Y , Lu XR , et al. Correlation between molecular prognostic factors and magnetic resonance imaging intravoxel incoherent motion histogram parameters in breast cancer. Magn Reson Imaging. 2022;85:262‐270. doi:10.1016/j.mri.2021.10.027 34740800

[30] Houssami N , Macaskill P , von Minckwitz G , Marinovich ML , Mamounas E . Meta‐analysis of the association of breast cancer subtype and pathologic complete response to neoadjuvant chemotherapy. Eur J Cancer. 2012;48(18):3342‐3354. doi:10.1016/j.ejca.2012.05.023 22766518

[31] Park SH , Moon WK , Cho N , et al. Diffusion‐weighted MR imaging: pretreatment prediction of response to neoadjuvant chemotherapy in patients with breast cancer. Radiology. 2010;257(1):56‐63. doi:10.1148/radiol.10092021 20851939

[32] Gillies RJ , Balagurunathan Y . Perfusion MR imaging of breast cancer: insights using “habitat imaging”. Radiology. 2018;288(1):36‐37. doi:10.1148/radiol.2018180271 29714676

[33] Zhang Y , Chen J , Yang C , et al. Preoperative prediction of microvascular invasion in hepatocellular carcinoma using diffusion‐weighted imaging‐based habitat imaging. Eur Radiol. 2024;34(5):3215‐3225. doi:10.1007/s00330-023-10339-2 37853175

[34] Angelini G , Marini C , Iacconi C , et al. Magnetic resonance (MR) features in triple negative breast cancer (TNBC) vs receptor positive cancer (nTNBC). Clin Imaging. 2018;49:12‐16. doi:10.1016/j.clinimag.2017.10.016 29120811

[35] Chen H , Li W , Wan C , Zhang J . Correlation of dynamic contrast‐enhanced MRI and diffusion‐weighted MR imaging with prognostic factors and subtypes of breast cancers. Front Oncol. 2022;12:942943. doi:10.3389/fonc.2022.942943 35992872 PMC9389013

[36] Bae MS , Shin SU , Ryu HS , et al. Pretreatment MR imaging features of triple‐negative breast cancer: association with response to neoadjuvant chemotherapy and recurrence‐free survival. Radiology. 2016;281(2):392‐400. doi:10.1148/radiol.2016152331 27195438

[37] Gentile LF , Plitas G , Zabor EC , Stempel M , Morrow M , Barrio AV . tumor biology predicts pathologic complete response to neoadjuvant chemotherapy in patients presenting with locally advanced breast cancer. Ann Surg Oncol. 2017;24(13):3896‐3902. doi:10.1245/s10434-017-6085-y 28916978 PMC5697706

[38] Antunovic L , De Sanctis R , Cozzi L , et al. PET/CT radiomics in breast cancer: promising tool for prediction of pathological response to neoadjuvant chemotherapy. Eur J Nucl Med Mol Imaging. 2019;46(7):1468‐1477. doi:10.1007/s00259-019-04313-8 30915523

