# Supplementary material for: Breast Cancer: Habitat imaging based on intravoxel incoherent motion for predicting pathologic complete response to neoadjuvant chemotherapy
Source: Med Phys. 2025 Apr 11;52(6):3711–22. doi: 10.1002/mp.17813 (PMC12149713; doi:10.1002/mp.17813)
Supplement: Supplementary file 2 — Supporting Information [file MP-52-3711-s002.docx]

**Supplement 1**

In this study, ERs and PRs are collectively referred to as hormone receptors (HRs). An HR-positive status was defined as ≥1% of tumor cell nuclei being positively stained for either ER or PR, while an HR-negative status was defined as <1% of tumor cell nuclei staining positive for both ER and PR. Tumors with an HER2 membrane immunostaining score of 3+ were considered to be positive for HER2 expression. If the membrane immunostaining score was 2+, in situ hybridization was performed to confirm HER2 amplification. Ki67 positivity was defined as ≥30% of tumor cell nuclei being positively stained for Ki67.

**Supplement 2**

The TCbHP regimen (carboplatin, docetaxel, pertuzumab, trastuzumab) was provided to 33 patients; the AC-THP regimen (trastuzumab, cyclophosphamide, paclitaxel, epirubicin, pertuzumab) was provided to 25 patients; the AC-T regimen (epirubicin, cyclophosphamide, paclitaxel) was provided to 23 patients; the TAC regimen (paclitaxel or docetaxel, epirubicin, cyclophosphamide) was provided to 22 patients; the AC regimen (epirubicin, cyclophosphamide) was provided to 24 patients; the AC-TP regimen (cyclophosphamide, epirubicin, paclitaxel, carboplatin) was provided to 6 patients; the TP regimen (paclitaxel, carboplatin) was provided to 4 patients; the AC-H regimen (epirubicin, cyclophosphamide, trastuzumab) was provided to 4 patients; the THP regimen (trastuzumab, paclitaxel, pertuzumab) was provided to 1 patient; and the AT regimen (epirubicin, paclitaxel) was provided to 1 patient.

**Supplement 3**

During preprocessing, we first used a re-segmentation strategy with 3 sigma restricted for D, f, and D*, respectively. Then, we discretized the images as a 16-bins and based on the whole training dataset, and used a 2.5 D merge strategy for the texture features. Finally, we extracted the following features: first-order, shape, gray-level co-occurrence matrix, gray-level run length matrix, gray-level size zone matrix, gray-level dependence matrix, and neighboring gray tone difference matrix. We also used a wavelet transform with a coif1 filter to extract more high-dimensional features. The entire feature-extraction process was conducted according to the Imaging Biomarker Standardization Initiative. We self-checked the radiomics quality score as follows:

Image protocol quality-well-documented image protocols (for example, contrast, slice thickness, energy, etc.) and/ or usage of public image protocols allow reproducibility/ replicability

☑ protocols well documented

□ public protocol used

□ none

Multiple segmentations-possible actions are: segmentation by different physicians/ algorithms/ software, perturbing segmentations by (random) noise, segmentation at different breathing cycles. Analyse feature robustness to segmentation variabilities

☑ yes

□ no

Phantom study on all scanners-detect inter-scanner differences and vendor-dependent features. Analyse feature robustness to these sources of variability

□ yes

☑ no

Imaging at multiple time points-collect images of individuals at additional time points. Analyse feature robustness to temporal variabilities (for example, organ movement, organ expansion/ shrinkage)

□ yes

☑ no

Feature reduction or adjustment for multiple testing-decreases the risk of overfitting. Overfitting is inevitable if the number of features exceeds the number of samples. Consider feature robustness when selecting features

☑ Either measure is implemented

□ Neither measure is implemented

Multivariable analysis with non radiomics features (for example, EGFR mutation) - is expected to provide a more holistic model. Permits correlating/ inferencing between radiomics and non radiomics features

☑ yes

□ no

Detect and discuss biological correlates-demonstration of phenotypic differences (possibly associated with underlying gene-protein expression patterns) deepens understanding of radiomics and biology

☑ yes

□ no

Cut-off analyse-determine risk groups by either the median, a previously published cut-off or report a continuous risk variable. Reduces the risk of reporting overly optimistic results

☑ yes

□ no

Discrimination statistics-report discrimination statistics (for example, C-statistic, ROC curve, AUC) and their statistical significance (for example, p-values, confidence intervals). One can also apply resampling method (for example, bootstrapping, cross-validation)

☑ a discrimination statistic and its statistical significance are reported

□ a resampling method technique is also applied

□ none

Calibration statistics-report calibration statistics (for example, Calibration-in-the-large/slope, calibration plots) and their statistical significance (for example, P-values, confidence intervals). One can also apply resampling method (for example, bootstrapping, cross-validation)

☑ a calibration statistic and its statistical significance are reported

□ a resampling method technique is applied

□ none

Prospective study registered in a trial database -provides the highest level of evidence supporting the clinical validity and usefulness of the radiomics biomarker

☑ yes

□ no

Validation-the validation is performed without retraining and without adaptation of the cut-off value, provides crucial information with regard to credible clinical performance

□ No validation

☑ validation is based on a dataset from the same institute

□ validation is based on a dataset from another institute

□ validation is based on two datasets from two distinct institutes

□ the study validates a previously published signature

□ validation is based on three or more datasets from distinct institutes

Comparison to 'gold standard'-assess the extent to which the model agrees with/is superior to the current 'gold standard' method (for example, TNM-staging for survival prediction). This comparison shows the added value of radiomics

☑ yes

□ no

Potential clinical utility-report on the current and potential application of the model in a clinical setting (for example, decision curve analysis).

☑ yes

□ no

Cost-effectiveness analysis-report on the cost-effectiveness of the clinical application (for example, QALYs generated)

☑ yes

□ no

Open science and data-make code and data publicly available. Open science facilitates knowledge transfer and reproducibility of the study

□ scans are open source

□ region of interest segmentations are open source

□ the code is open sourced

☑ radiomics features are calculated on a set of representative ROIs and the calculated features and representative ROIs are open source

Total score

25 (69.44%)

**Supplement 4**

1. We tried to determine the number of habitats using a bootstrap approch. The specific steps are as follows:

The data were randomly sampled repeatedly with replacement till sample size was the same as that of the original dataset. Then the sampled dataset was used to calculate the CH index. This process was repeated 50 times, and then the average and standard deviation of CH index were calculated. Then we applied the one standard error rule (1-SE) to select the minimum k that produced an average CH index greater then the maximum average CH index minus one standard error of the corresponding CH index (Figure 1). Furthermore, we also found the standard deviation of the CH index is minimum when k=3.


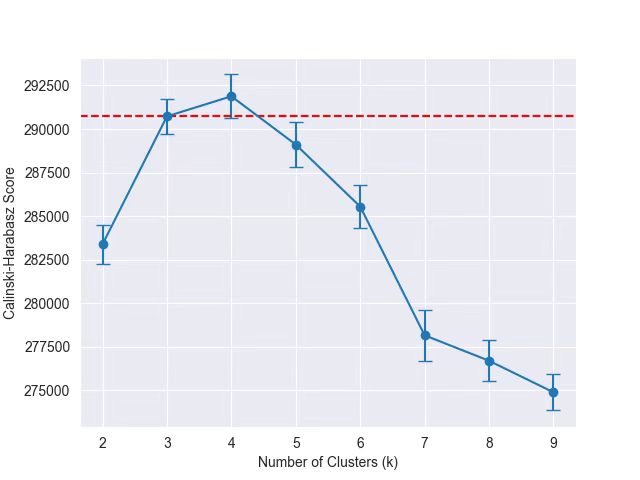


Figure 1: The plot of the Calinski-Harabasz score against to the cluster K number

1. As shown in the scatter plots, when K = 4 was used, the meaning of part 4 was more difficult to explain than that of the other parts. On the other hand, when K = 3 was used, each part had a clear meaning (Figure 2).


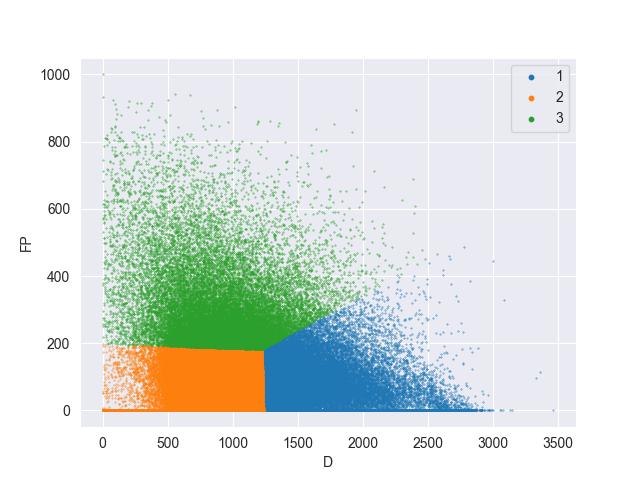

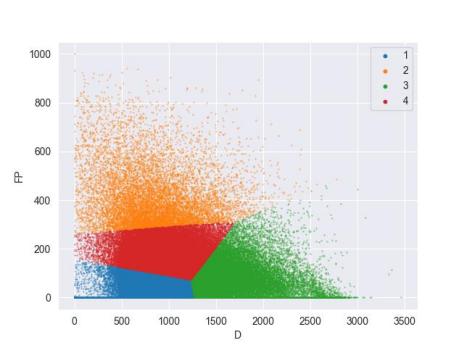


Figure 2: The scatter plots (left: K = 3; right: K = 4)

**Supplement 5**

The models were built based on scikit-learn package and the unmentioned hyper-parameters were set as default., as follows:

1.Hyperparameter Configuration and Tuning for LR:

A cross-validation grid-search approach was used to determine the best combinations of the hyper-parameters in the following scope:

-C: (0.01, 0.1, 1.0, 5)

-tol: (0.00003, 0.0003, 0.003)

-max_iter: (100, 200)

2.Hyperparameter Configuration and Tuning for SVM:

A cross-validation grid-search approach was used to determine the best combinations of the hyper-parameters in the following scope:

-Linear Kernel (kernel= ‘linear’): Optimize C as [0.1, 0.3, 1.0, 3.0].

-RBF Kernel (kernel=’rbf’): Jointly optimize gamma as [0.1, 0.3, 1.0, 3.0].

-Polynomial Kernel (kernel=’poly’): Test degree as [2, 3, 4].

**Supplement 6**

Table 2: Results of ROC curve analysis of different parameter combinations

| Starting feature set | Final feature set (weight) | |
| --- | --- | --- |
| **Whole-tumor radiomics** | |  |
| Shape features, first-order features, second-order features, and texture features, including GrayLevelCooccurence Matrix (GLCM), Gray LevelRun Length Matrix (GLRLM), Gray LevelSize Zone Matrix (GLSZM), Neighbouring Gray Tone Difference Matrix (NGTDM), Gray Level Dependence Matrix (GLDM) from D and F maps | IVIM_D_wavelet-LHL_glszm_GrayLevelNonUniformityNormalized (-1.111)  IVIM_D_LoG2-3D_gldm_LargeDependenceLowGrayLevelEmphasis (-0.312)  IVIM_D_LoG3-3D_glrlm_ShortRunLowGrayLevelEmphasis (0.326)  IVIM_D_LoG3-3D_glszm_SizeZoneNonUniformityNormalized (0.354)  IVIM_D_wavelet-LHH_glcm_ClusterTendency (0.386)  IVIM_D_wavelet-LHH_glcm_Imc2 (0.448)  IVIM-f_wavelet-LLH_glcm_Id (0.059)  IVIM-f_wavelet-LLH_ngtdm_Contrast (0.101)  IVIM-f_wavelet-HLH_glcm_Imc2 (0.374) | |
| **Habitat analysis** |  | |
| D_mask and IVIM-f_mask  (10Percentile, 90Percentile, Energy, Entropy, InterquartileRange, Kurtosis, Maximum, MeanAbsoluteDeviation, Mean, Median, Minimum, Range, RobustMeanAbsoluteDeviation, RootMeanSquared, Skewness, TotalEnergy, Uniformity, Variance, Volume)  IVIM-D(IVIM-f)_mask_Percent | IVIM-f_mask1_10Percentile (0.851)  IVIM-D_mask2_Entropy (-0.131)  IVIM-D_mask2_Range (-0.448)  IVIM-D_mask2_Uniformity (0.234)  IVIM-D_mask3_Minimum (-0.414)  IVIM-D_mask3_Skewness (-0.429)  IVIM-D_mask3_Uniformity (-0.384)  IVIM-f_mask3_10Percentile (0.177)  IVIM-f_mask3_Minimum (-0.008) | |
| **Conventional MRI features** | |  |
| Age  FGT  BPE level  Location  Masses or NME  Irregular  Spiculated  Rim enhancement  Clustered ring  Architectural distortion  T2 high signal  TIC | | Rim enhancement (0.537)  T2 high signal (-0.698) |
| **Immunohistochemistry** | |  |
| HR  HER2  Ki-67  Lymph nodes | | HR (-0.412)  HER2 (1.241) |

| **Habitat + Conventional MRI features** |  | |
| --- | --- | --- |
| IVIM-f_mask1_10PercentileIVIM-D_mask2_Entropy  IVIM-D_mask2_Range  IVIM-D_mask2_Uniformity  IVIM-D_mask3_Minimum  IVIM-D_mask3_Skewness  IVIM-D_mask3_Uniformity  IVIM-f_mask3_10Percentile  IVIM-f_mask3_Minimum  Rim enhancement  T2 high signal | | IVIM-f_mask1_10Percentile (0.901)  IVIM-D_mask2_Entropy (-0.214)  IVIM-D_mask2_Range (-0.390)  IVIM-D_mask2_Uniformity (0.175)  IVIM-D_mask3_Minimum (-0.355)  IVIM-D_mask3_Skewness (-0.586)  IVIM-D_mask3_Uniformity (-0.649)  IVIM-f_mask3_10Percentile (0.185)  IVIM-f_mask3_Minimum (0.027)  Rim enhancement (0.510)  T2 high signal (-0.857) |
| **Whole-tumor radiomics + Conventional MRI features** |  | |
| IVIM_D_wavelet-LHL_glszm_GrayLevelNonUniformityNormalized  IVIM_D_LoG2-3D_gldm_LargeDependenceLowGrayLevelEmphasis  IVIM_D_LoG3-3D_glrlm_ShortRunLowGrayLevelEmphasis  IVIM_D_LoG3-3D_glszm_SizeZoneNonUniformityNormalized  IVIM_D_wavelet-LHH_glcm_ClusterTendency  IVIM_D_wavelet-LHH_glcm_Imc2  IVIM-f_wavelet-LLH_glcm_Id  IVIM-f_wavelet-LLH_ngtdm_Contrast  IVIM-f_wavelet-HLH_glcm_Imc2  Rim enhancement  T2 high signal | | IVIM_Dwavelet-LHL_glszm_GrayLevelNonUniformityNormalized (-1.173)  IVIM_DLoG3-3D_glrlm_ShortRunLowGrayLevelEmphasis (0.685)  IVIM_DLoG3-3D_glszm_SizeZoneNonUniformityNormalized (0.584)  IVIM_Dwavelet-LHH_glcm_ClusterTendency (1.031)  IVIM_Dwavelet-LHH_glcm_Imc2 (1.037)  IVIM-fwavelet-LHL_firstorder_Median (0.794)  Rim enhancement (0.874)  T2 high signal (-1.467) |

| **Conventional MRI features + Immunohistochemistry** |  | |
| --- | --- | --- |
| Rim enhancement  T2 high signal  HR, HER2 | | Rim enhancement (0.413)  T2 high signal (-1.082)  HR (-0.622)  HER2 (1.298) |
| **Habitat + Conventional MRI features + Immunohistochemistry** | | |
| IVIM-f_mask1_10PercentileIVIM-D_mask2_Entropy  IVIM-D_mask2_Range  IVIM-D_mask2_Uniformity  IVIM-D_mask3_Minimum  IVIM-D_mask3_Skewness  IVIM-D_mask3_Uniformity  IVIM-f_mask3_10Percentile  IVIM-f_mask3_Minimum  Rim enhancement, T2 high signal, HR, HER2 | | IVIM-f_mask1_10Percentile (1.114)  IVIM-D_mask2_Uniformity (0.348)  IVIM-f_mask3_Minimum (-0.148)  T2 high signal (-0.848)  HR (-0.618)  HER2 (1.399) |
| **Whole-tumor radiomics + Conventional MRI features + Immunohistochemistry** | | |
| IVIM_Dwavelet-LHL_glszm_GrayLevelNonUniformityNormalized  IVIM_DLoG3-3D_glrlm_ShortRunLowGrayLevelEmphasis  IVIM_DLoG3-3D_glszm_SizeZoneNonUniformityNormalized  IVIM_Dwavelet-LHH_glcm_ClusterTendency  IVIM_Dwavelet-LHH_glcm_Imc2  IVIM-fwavelet-LHL_firstorder_Median  Rim enhancement  T2 high signal, HR, HER2 | | IVIM_Dwavelet-LHL_glszm_GrayLevelNonUniformityNormalized (-0.650)  IVIM_Dwavelet-LHH_glcm_ClusterTendency (0.193)  IVIM_Dwavelet-LHH_glcm_Imc2 (0.858)  T2 high signal (-0.936)  HR (-0.530)  HER2 (1.323) |
