# Supplementary material for: Breast Cancer: Habitat imaging based on intravoxel incoherent motion for predicting pathologic complete response to neoadjuvant chemotherapy
Source: Med Phys. 2025 Apr 11;52(6):3711–22. doi: 10.1002/mp.17813 (PMC12149713; doi:10.1002/mp.17813)
Supplement: Supplementary file 4 — Supporting Information [file MP-52-3711-s003.pdf]

Supplement 1

In this study, ERs and PRs are collectively referred to as hormone receptors (HRs). An HR-positive status was defined as ≥1% of tumor cell nuclei being positively stained for either ER or PR, while an HR-negative status was defined as <1% of tumor cell nuclei staining positive for both ER and PR. Tumors with an HER2 membrane immunostaining score of 3+ were considered to be positive for HER2 expression. If the membrane immunostaining score was 2+, in situ hybridization was performed to confirm HER2 amplification. Ki67 positivity was defined as ≥30% of tumor cell nuclei being positively stained for Ki67.

Supplement 2

The TCbHP regimen (carboplatin, docetaxel, pertuzumab, trastuzumab) was provided to 33 patients; the AC-THP regimen (trastuzumab, cyclophosphamide, paclitaxel, epirubicin, pertuzumab) was provided to 25 patients; the AC-T regimen (epirubicin, cyclophosphamide, paclitaxel) was provided to 23 patients; the TAC regimen (paclitaxel or docetaxel, epirubicin, cyclophosphamide) was provided to 22 patients; the AC regimen (epirubicin, cyclophosphamide) was provided to 24 patients; the AC-TP regimen (cyclophosphamide, epirubicin, paclitaxel, carboplatin) was provided to 6 patients; the TP regimen (paclitaxel, carboplatin) was provided to 4 patients; the AC-H regimen (epirubicin, cyclophosphamide, trastuzumab) was provided to 4 patients; the THP regimen (trastuzumab, paclitaxel, pertuzumab) was provided to 1 patient; and the AT regimen (epirubicin, paclitaxel) was provided to 1 patient.

Supplement 3

During preprocessing, we first used a re-segmentation strategy with 3 sigma restricted for D, f, and D*, respectively. Then, we discretized the images as a 16-bins and based on the whole training dataset, and used a 2.5 D merge strategy for the texture features. Finally, we extracted the following features: first-order, shape, gray-level co-occurrence matrix, gray-level run length matrix, gray-level size zone matrix, gray-level dependence matrix, and neighboring gray tone difference matrix. We also used a wavelet transform with a coif1 filter to extract more high-dimensional features. The entire feature-extraction process was conducted according to the Imaging Biomarker Standardization Initiative. We self-checked the radiomics quality score as follows:

Image protocol quality-well-documented image protocols (for example, contrast, slice thickness, energy, etc.) and/ or usage of public image protocols allow reproducibility/ replicability

☑ protocols well documented

☐ public protocol used

☐ none

Multiple segmentations-possible actions are: segmentation by different physicians/ algorithms/ software, perturbing segmentations by (random) noise, segmentation at different breathing cycles. Analyse feature robustness to segmentation variabilities

☑ yes

☐ no

Phantom study on all scanners-detect inter-scanner differences and vendor-dependent features. Analyse feature robustness to these sources of variability

☐ yes

☑ no

Imaging at multiple time points-collect images of individuals at additional time points. Analyse feature robustness to temporal variabilities (for example, organ movement, organ expansion/ shrinkage)

☐ yes

☑ no

Feature reduction or adjustment for multiple testing-decreases the risk of overfitting. Overfitting is inevitable if the number of features exceeds the number of samples. Consider feature robustness when selecting features

☑ Either measure is implemented

☐ Neither measure is implemented

Multivariable analysis with non radiomics features (for example, EGFR mutation) - is expected to provide a more holistic model. Permits correlating/ inferencing between radiomics and non radiomics features

☑ yes

☐ no

Detect and discuss biological correlates-demonstration of phenotypic differences (possibly associated with underlying gene-protein expression patterns) deepens understanding of radiomics and biology

☑ yes

☐ no

Cut-off analyse-determine risk groups by either the median, a previously published cut-off or report a continuous risk variable. Reduces the risk of reporting overly optimistic results

☑ yes

☐ no

Discrimination statistics-report discrimination statistics (for example, C-statistic, ROC curve, AUC) and their statistical significance (for example, p-values, confidence intervals). One can also apply resampling method (for example, bootstrapping, cross-validation)

☑ a discrimination statistic and its statistical significance are reported

☐ a resampling method technique is also applied

☐ none

Calibration statistics-report calibration statistics (for example, Calibration-in-the-large/slope, calibration plots) and their statistical significance (for example, P-values, confidence intervals). One can also apply resampling method (for example, bootstrapping, cross-validation)

☑ a calibration statistic and its statistical significance are reported

☐ a resampling method technique is applied

☐ none

Prospective study registered in a trial database -provides the highest level of evidence

supporting the clinical validity and usefulness of the radiomics biomarker

☑ yes

☐ no

Validation-the validation is performed without retraining and without adaptation of the cut-off value, provides crucial information with regard to credible clinical performance

☐ No validation

☑ validation is based on a dataset from the same institute

☐ validation is based on a dataset from another institute

☐ validation is based on two datasets from two distinct institutes

☐ the study validates a previously published signature

☐ validation is based on three or more datasets from distinct institutes

Comparison to 'gold standard'-assess the extent to which the model agrees with/is superior to the current 'gold standard' method (for example, TNM-staging for survival prediction). This comparison shows the added value of radiomics

☑ yes

☐ no

Potential clinical utility-report on the current and potential application of the model in a clinical setting (for example, decision curve analysis).

☑ yes

☐ no

Cost-effectiveness analysis-report on the cost-effectiveness of the clinical application

(for example, QALYs generated)

☑ yes

☐ no

Open science and data-make code and data publicly available. Open science facilitates knowledge transfer and reproducibility of the study

☐ scans are open source

☐ region of interest segmentations are open source

☐ the code is open sourced

☑ radiomics features are calculated on a set of representative ROIs and the calculated features and representative ROIs are open source

Total score

25 (69.44%)
